# Coordination of asparagine uptake and asparagine synthetase expression modulates CD8^+^ T cell activation

**DOI:** 10.1172/jci.insight.137761

**Published:** 2021-05-10

**Authors:** Helen Carrasco Hope, Rebecca J. Brownlie, Christopher M. Fife, Lynette Steele, Mihaela Lorger, Robert J. Salmond

**Affiliations:** Leeds Institute of Medical Research at St. James’s, University of Leeds, Wellcome Trust Brenner Building, St. James’s University Hospital, Leeds, United Kingdom.

**Keywords:** Immunology, Amino acid metabolism, T cells

## Abstract

T cell receptor (TCR) triggering by antigen results in metabolic reprogramming that, in turn, facilitates the exit of T cells from quiescence. The increased nutrient requirements of activated lymphocytes are met, in part, by upregulation of cell surface transporters and enhanced uptake of amino acids, fatty acids, and glucose from the environment. However, the role of intracellular pathways of amino acid biosynthesis in T cell activation is relatively unexplored. Asparagine is a nonessential amino acid that can be synthesized intracellularly through the glutamine-hydrolyzing enzyme asparagine synthetase (ASNS). We set out to define the requirements for uptake of extracellular asparagine and ASNS activity in CD8^+^ T cell activation. At early time points of activation in vitro, CD8^+^ T cells expressed little or no ASNS, and, as a consequence, viability and TCR-stimulated growth, activation, and metabolic reprogramming were substantially impaired under conditions of asparagine deprivation. At later time points (more than 24 hours of activation), TCR-induced mTOR-dependent signals resulted in ASNS upregulation that endowed CD8^+^ T cells with the capacity to function independently of extracellular asparagine. Thus, our data suggest that the coordinated upregulation of ASNS expression and uptake of extracellular asparagine is involved in optimal T cell effector responses.

## Introduction

Naive T lymphocytes are small quiescent cells that take up low levels of nutrients to sustain a basal catabolic metabolism. By contrast, antigenic stimulation of T cells results in the upregulation of nutrient receptors and the engagement of anabolic processes to generate the biomolecules required for rapid growth, proliferation, and effector function. In recent years, critical roles in T cell activation and differentiation have been determined for upregulated expression of the heterodimeric neutral amino acid transporter Slc7a5/Slc3a2 and glutamine (Gln) transporter Slc1a5 (1, 2). Furthermore, uptake of a number of amino acids, including Gln (2, 3), leucine (4, 5), tryptophan (6), and methionine (7), have been determined to play important functions in T cell activation. Despite the fact that a number of key amino acids, such as Gln, serine, and alanine, can be synthesized intracellularly, extracellular sources of these amino acids are required for T cells to meet their metabolic requirements upon exit from quiescence and to facilitate activation (8, 9).

The uptake and metabolism of amino acids play a key role in numerous intracellular processes in lymphocytes. For example, Gln is used as a building block for protein synthesis, to maintain activation of the mechanistic target of rapamycin complex 1 (mTORC1) (2), to replenish Krebs cycle intermediates via glutaminolysis (10), to synthesize glutathione (11), and to fuel posttranslational *O*-GlcNAcylation of key proteins (12). A further potential fate for Gln is the production of asparagine via the activity of the Gln-hydrolyzing enzyme asparagine synthetase (ASNS) (13). Interestingly, asparagine metabolism and uptake have emerged as a key target in cancer therapy. Thus, ASNS expression is a predictive biomarker in several human cancer types (14, 15) whereas asparaginases, that degrade extracellular asparagine, are a key chemotherapeutic drug for the treatment of leukemia and lymphomas that lack ASNS expression (13). Furthermore, upregulation of ASNS expression via the stress-induced activating transcription factor 4 (ATF4) occurs in B and T cells in response to glucose and amino acid deprivation (16, 17). However, to date, the physiological role of intracellular asparagine production via ASNS in T cell responses is unknown. Bacterial asparaginases act to suppress T cell activation (18, 19), implying that T cells require extracellular sources of asparagine. However, asparaginases frequently have glutaminase activity, and asparaginases that lack this function are less immunosuppressive in mice (20).

In the current work, we set out to determine roles for extracellular asparagine uptake and intracellular asparagine production via ASNS in T cell activation. We show that, in naive mouse CD8^+^ T cells, ASNS expression is very low or absent, and asparagine uptake from the extracellular environment is required for maintenance of cell viability, initial stages of T cell growth, and activation in vitro. Furthermore, under conditions of asparagine deprivation, metabolic switching is compromised, likely as a consequence of defective T cell activation, resulting in decrease nutrient uptake, reduced glycolytic flux, oxidative phosphorylation (OXPHOS), and ATP production. T cell receptor (TCR) triggering and metabolic reprogramming via mTORC1 and Myc activation are accompanied by increased expression of ASNS, and subsequently CD8^+^ T cells can function independently of extracellular sources of asparagine. Using gene-trap mice expressing a hypomorphic *Asns* allele, we determined that ASNS expression is essential for in vitro CD8^+^ T cell activation in the absence of extracellular asparagine. Together, these data suggest an important role for coordinated asparagine uptake and biosynthesis in T cell activation and effector responses.

## Results

### Asparagine uptake is required for initial cell growth, survival, and activation following TCR stimulation.

**Figure 1:**
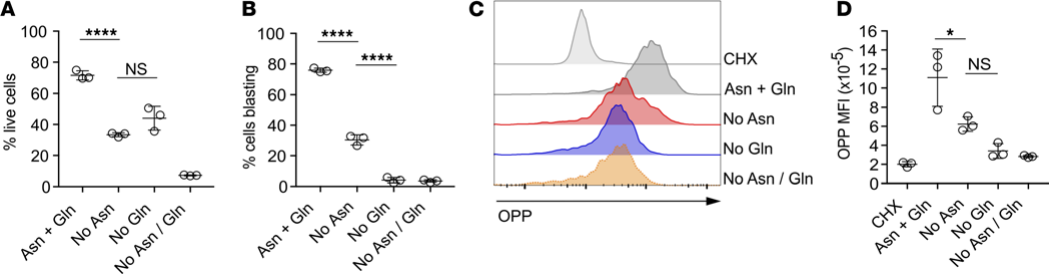
Extracellular asparagine is required to maintain viability and initiate cell growth following T cell receptor stimulation. OT-1 T cell receptor (TCR) transgenic T cells were stimulated with cognate SIINFEKL peptide for 24 hours in DMEM supplemented with or without asparagine and Gln, as indicated. (**A**) T cell viability was assessed by exclusion of Live/Dead Aqua dyes and FACS. (**B**) T cell growth was assessed by analysis of forward scatter and side scatter area (FSC-A/SSC-A) parameters on gated live cells by flow cytometry. (**C**) Nascent protein synthesis was assessed by incorporation of OPP, intracellular staining, and labeling using Click chemistry reagents and FACS analysis. Cycloheximide (CHX) was used as a negative control. (**D**) O-propargyl-puromycin (OPP) mean fluorescence intensity (MFI) was assessed by FACS analysis. Data are from 1 of 3 experiments, and individual data points (*n* = 3) represent technical replicates. *P < 0.05, ****P < 0.0001, as assessed by 1-way ANOVA, with Tukey’s multiple comparisons test.

We tested the effect of asparagine and/or Gln deprivation on TCR-induced cell growth and activation. Naive TCR transgenic OT-I CD8^+^ T cells were stimulated for 24 hours with cognate peptide SIINFEKL in DMEM supplemented with or without asparagine and Gln. Deprivation of either asparagine or Gln alone reduced, whereas the combined absence of both amino acids substantially impaired, T cell viability (Figure 1A). Most (70%–80%) of the T cells cultured in the presence of both amino acids had initiated blastogenesis following 24 hours of TCR stimulation (Figure 1B). By contrast, OT-I T cells stimulated with peptide in the absence of Gln or the combined absence of Gln and asparagine did not form a blast cell population (Figure 1B). An intermediate phenotype was determined for OT-I cells activated in the absence of asparagine alone; a reduced but reproducible proportion (20%–40%) of the cells had a blast phenotype (Figure 1B). A fundamental role for amino acids is to act as building blocks for protein synthesis. Consistent with the effect on T cell growth, we determined that levels of protein synthesis, as measured by assessment of uptake and incorporation of O-propargyl-puromycin (OPP), by TCR-stimulated OT-I T cells were substantially reduced in the absence of extracellular asparagine and/or Gln (Figure 1, C and D).

**Figure 2:**
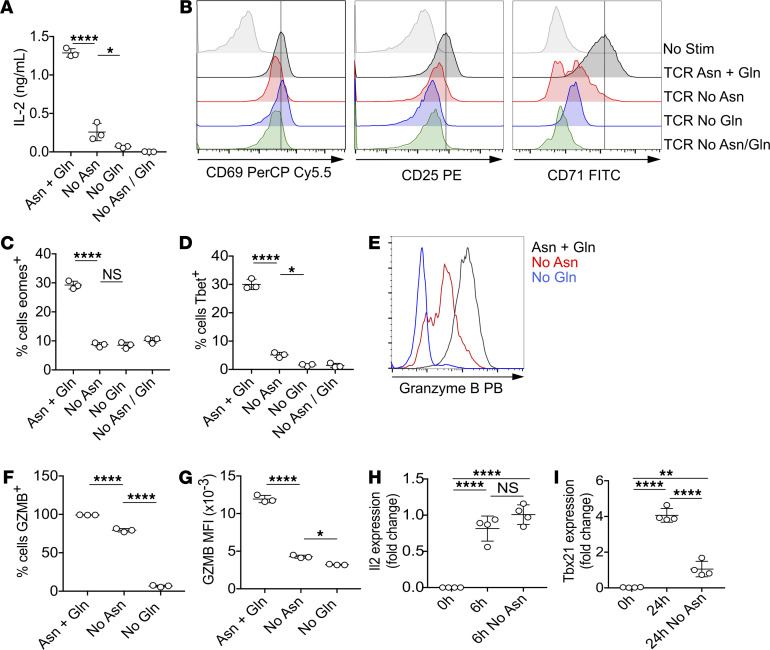
Extracellular asparagine is limiting for initial TCR-induced T cell activation. OT-1 TCR transgenic T cells were stimulated with cognate SIINFEKL peptide for 6 (**H**), 24 (**A–D**, and **I**), or 48 hours (**E–G**) in DMEM supplemented with or without asparagine and/or Gln, as indicated. (**A**) Levels of IL-2 in culture supernatants were assessed by ELISA. (**B**) Histograms show levels of cell surface activation marker expression on live-gated cells. Data are representative of 1 of at least 3 repeated experiments. Proportions of live-gated cells expressing transcriptions factors eomesodermin (eomes) (**C**) and Tbet (**D**) were determined by intracellular staining and FACS. (**E**) Representative histograms show levels of intracellular granzyme B in live-gated cells. Proportions of granzyme B^+^ (GZMB^+^) cells (**F**) were determined. Relative levels of expression of GZMB in live GZMB^+^ cells were assessed as mean fluorescence intensity (MFI) (**G**). Levels of *Il2* (**H**) and *Tbx21* (**I**) transcripts were determined by qRT-PCR. Levels are fold change using the ddCT method and *Rpl13a* as a housekeeping gene. For **H** and **I**, dots represent biological replicates (*n* = 4) from 1 of 2 repeated experiments. For all other data, dots represent technical replicates (*n* = 3) from at least 3 repeated experiments. *P < 0.05, **P < 0.01, ****P < 0.0001, as assessed by 1-way ANOVA, with Tukey’s multiple comparisons test.

Next, we assessed the effect of amino acid deprivation on TCR-induced activation marker and cytokine expression. Antigen-stimulated OT-I T cell IL-2 secretion was inhibited 70%–90% by deprivation of asparagine and/or Gln (Figure 2A). Additionally, although TCR-induced upregulation of the very early activation marker CD69 was unimpeded, cell surface expression of CD25 and transferrin receptor CD71 was partially or completely inhibited by a lack of extracellular asparagine and/or Gln (Figure 2B). Similarly, the proportions of T cells expressing key transcription factors eomesodermin (Figure 2C) and T-box expressed in T cells (Tbet) (Figure 2D) were reduced under conditions of asparagine or Gln deprivation. TCR-induced expression of cytolytic effector protein granzyme B was low under all media conditions after 24 hours of stimulation. Asparagine deprivation impaired TCR-induced granzyme B upregulation, as assessed following 48 hours of stimulation (Figure 2E). Both proportions of positive cells (Figure 2F) and levels of granzyme B within positive cells, as assessed by mean fluorescence intensity (Figure 2G), were reduced compared with control conditions. As compared with either asparagine deprivation or control conditions, Gln deprivation more severely impaired TCR-induced granzyme B expression (Figure 2, E–G).

A key question was whether the absence of extracellular asparagine impinged on the expression of T cell activation markers and effector proteins was simply a consequence of decreased protein synthesis or also affected gene transcription. To this end, we assessed transcript levels of *Il2* and *Tbx21* (encoding Tbet) following activation of OT-I T cells in the presence or absence of asparagine for 6 and 24 hours, respectively, by quantitative reverse transcriptase-PCR (qRT-PCR). Data indicated that early upregulation of *Il2* expression was unimpaired in the absence of asparagine (Figure 2H), indicating that decreased IL-2 secretion was a consequence of posttranscriptional effects, most likely decreased protein synthesis. By contrast, *Tbx21* levels were much reduced under conditions of asparagine deprivation (Figure 2I), indicating that decreased transcript levels contribute to the effects on Tbet expression.

IL-2 is a potent T cell growth factor and mitogen. Thus, it was possible that a failure to produce IL-2 was responsible for reduced OT-I T cell viability, growth, and activation under conditions of amino acid deprivation. However, addition of exogenous recombinant IL-2 did not rescue T cell viability, protein synthesis, or enhance TCR-induced expression of granzyme B in asparagine- or Gln-deprived media (Supplemental Figure 1; supplemental material available online with this article; https://doi.org/10.1172/jci.insight.137761DS1). Taken together, these data indicate that CD8^+^ T cells require extracellular sources of asparagine and Gln to maintain cell viability and initiate cell growth, protein synthesis, and activation following TCR stimulation. Either amino acid alone is sufficient to maintain a reduced proportion of viable cells; however, the combined absence of asparagine and Gln results in catastrophic levels of cell death.

### Asparagine availability regulates TCR-induced metabolic reprogramming.

**Figure 3:**
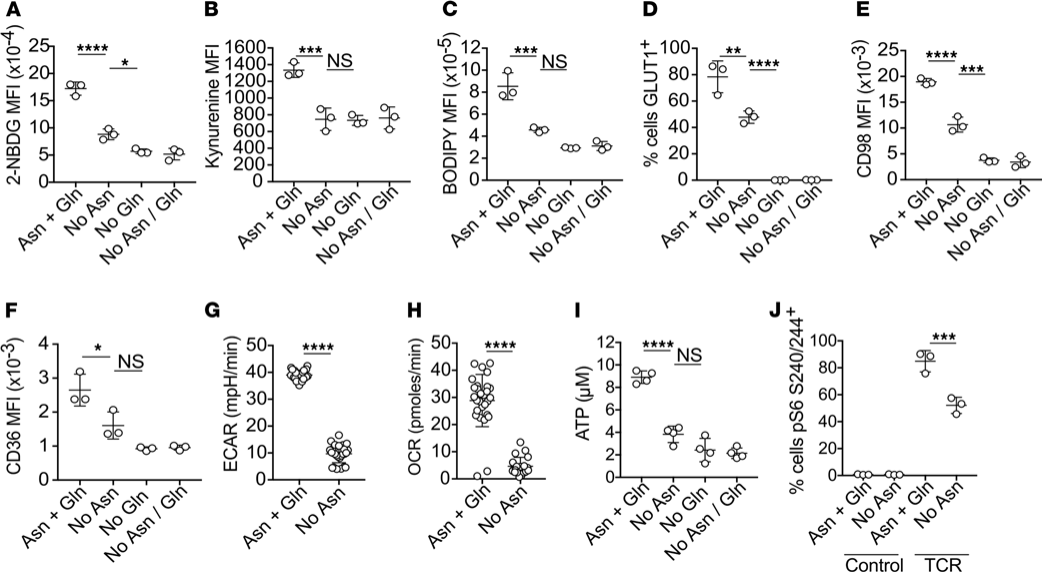
Extracellular asparagine is limiting for initial TCR-induced metabolic reprogramming. OT-1 T cells were stimulated with cognate SIINFEKL peptide for 24 hours in DMEM supplemented with or without asparagine and/or Gln, as indicated. Uptake of fluorescent glucose analogue 2-NBDG (**A**), kynurenine (**B**), and long-chain fatty acid BODIPY-C16 (**C**), and cell surface expression of transporters GLUT1 (**D**), CD98 (**E**), and CD36 (**F**) were assessed by FACS analysis. Data are mean fluorescence intensity values (MFI) or percentage of cells positive, as indicated. (**G** and **H**). Extracellular acidification rate (ECAR) and oxygen consumption rate (OCR) of activated OT-1 T cells were assessed using a Seahorse metabolic analyzer. (**I**) ATP production was assessed using a luminescence-based assay and luminometer. (**J**) Proportions of phospho-ribosomal protein S6^+^ (pS6 S240/244^+^) cells were assessed by intracellular staining and FACS analysis. Data are representative of 2 (**G** and **H**) or 3 repeated experiments. Data points represent technical replicates; (**A–F**, and **J**) *n* = 3, (**G** and **H**) *n* = 30, and (**I**) *n* = 4. *P < 0.05, **P < 0.01, ***P < 0.001, ****P < 0.0001, as determined by 1-way ANOVA, with Tukey’s multiple comparisons test (**A–F**, and **I**), 2-tailed Student’s *t* test (**G** and **H**), or 2-way ANOVA (**J**).

Nutrient uptake and metabolic reprogramming following TCR stimulation are highly coordinated. Therefore, it was of interest to determine whether the availability of asparagine impinged upon T cell metabolism. The tryptophan metabolite kynurenine is transported into T cells via the neutral amino acid transporter Slc7a5 (also known as CD98) (21), which also serves as a transporter for essential amino acids tryptophan, leucine, phenylalanine, and methionine. We took advantage of the fluorescent properties of kynurenine as well as the glucose analog 2-NB-d-glucose (2-NBDG) and long-chain fatty acid analog 4, 4-difluoro-5, 7-dimethyl-4-bora-3a, 4a-diaza-s-indacene-3-hexadecanoic acid (BODIPY) C16 to assess the effect of amino acid deprivation on TCR-induced nutrient uptake. FACS analysis demonstrated that OT-I T cells activated for 24 hours in the absence of asparagine and/or Gln had reduced capacity to uptake glucose (Figure 3A), kynurenine (Figure 3B), and BODIPY C16 (Figure 3C) as compared with cells activated in the presence of both amino acids. Reduced TCR-induced nutrient uptake was associated with impaired cell surface expression of nutrient transporters GLUT1 (also known as Slc2a1), CD98, and CD36 under conditions of asparagine with or without Gln deprivation (Figure 3, D–F).

Analysis of extracellular acidification rate (ECAR) and oxygen consumption rate (OCR) serve as proxy measures for lactate secretion/glycolytic rate and OXPHOS, respectively. Seahorse metabolic analysis demonstrated that OT-I T cells activated under conditions of asparagine deprivation had reduced basal ECAR (Figure 3G) and OCR (Figure 3H). To assess mitochondrial function in more detail, we performed a Mitostress test. Data indicated that cells activated in the absence of asparagine had substantially reduced basal and stressed OCR (Supplemental Figure 2A). Despite reduced OCR of asparagine-deprived T cells, their basal OCR/ECAR ratio was consistently higher than cells activated in asparagine-replete media (Supplemental Figure 2B), consistent with a failure to effectively engage glycolytic pathways and suggestive of overall reduced levels of cellular metabolism. Consistent with these data, levels of OT-I T cell ATP production were reduced by 50%–75% in asparagine- and/or Gln-deprived media (Figure 3I).

The availability of amino acids, including Gln, has previously been shown to play a central role in T cell activation and metabolism through regulation of the activity of the mTORC1 kinase complex (1, 2). Analysis of phosphorylation of the downstream substrate ribosomal protein S6 (22) determined that, in the absence of extracellular asparagine, TCR-induced mTORC1 activation was impaired but not blocked (Figure 3J). Taken together, these data indicate that, at initial stages of T cell activation, the bioavailability of asparagine is essential for T cell activation and subsequently determines the extent of TCR-induced nutrient uptake, glycolytic flux, OXPHOS, and energy production, with a partial effect on mTORC1 activation.

### CD8^+^ T cells lose requirement for extracellular asparagine upon prolonged TCR stimulation.

**Figure 4:**
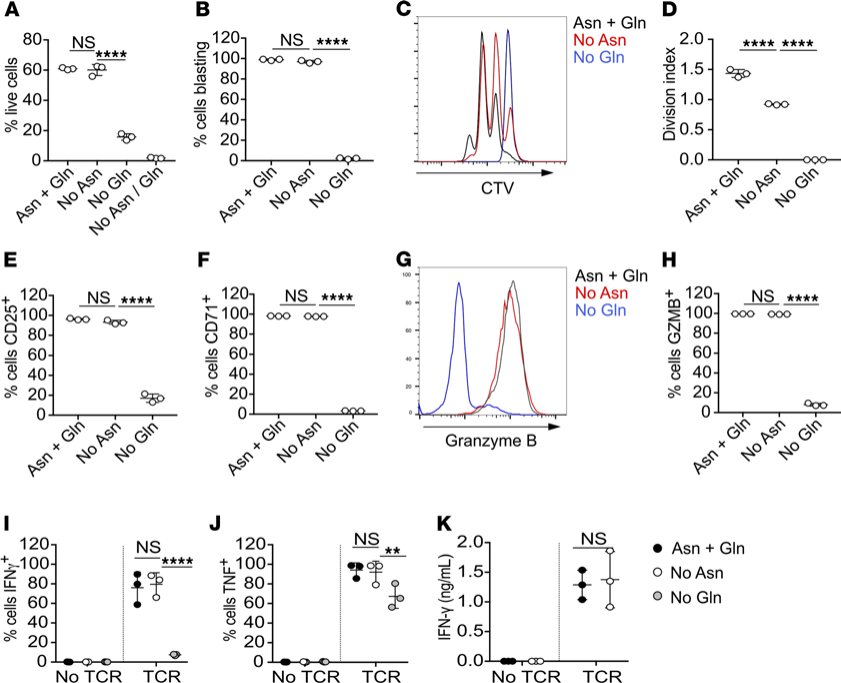
T cells lose requirement for extracellular asparagine upon prolonged activation. OT-1 T cells were stimulated with cognate SIINFEKL peptide for 72 hours (**A–G**) or were differentiated for 6 days (**H** and **I**) in DMEM supplemented with or without asparagine and/or Gln or complete IMDM (**J** and **K**), as indicated. T cell viability (**A**) and proportions of blasting cell populations (**B**) were assessed by exclusion of Live/Dead Aqua dyes and analysis of forward scatter and side scatter area (FSC-A/SSC-A) parameters by flow cytometry, respectively. For analysis of TCR-induced proliferation, cells were labeled with Cell Trace Violet (CTV) prior to stimulation. Histograms are representative of data from 2 repeated experiments (**C**). Division indices were calculated using FlowJo software (**D**). Proportions of live-gated T cells expressing CD25 (**E**), CD71 (**F**), or granzyme B (**G** and **H**) were determined by flow cytometry. (**I** and **J**) Effector cytotoxic T lymphocytes (CTLs) were differentiated for 6 days in DMEM with or without asparagine/Gln, then restimulated in complete IMDM for 4 hours. Proportion of IFN-γ^+^ and TNF^+^ cells were determined by intracellular staining and FACS analysis. (**K**) Effector CTLS were differentiated in complete IMDM for 5 days, before a 24-hour washout in DMEM with or without asparagine, then were restimulated in DMEM with or without asparagine for 24 hours. IFN-γ levels in supernatants were determined by ELISA. Data are representative of 2 (**C** and **D**) or 3 repeated experiments. In all graphs, individual data points represent technical replicates (*n* = 3). **P < 0.01, ****P < 0.0001, as determined by 1-way ANOVA with Tukey’s multiple comparisons test (**A**, **B**, **D–F**, and **H**) or 2-way ANOVA with Sidak’s multiple comparisons test (**I–K**).

Notably, at early time points (24 hours), in contrast to Gln deprivation, a reduced but consistent proportion of OT-I T cells stimulated in the absence of extracellular asparagine demonstrated activation profiles comparable to cells stimulated under control conditions (i.e., blast phenotype, elevated activation marker expression/OPP incorporation, and phospho-S6^+^) (Figures 1–3). We sought to determine whether T cell activation remained dependent on extracellular asparagine at later time points. Following 72 hours of peptide stimulation, levels of OT-I cell viability were comparable in asparagine-deprived conditions as compared with control media conditions (Figure 4A). By contrast, OT-I T cell viability was much impaired under conditions of Gln deprivation (10%–20%), whereas T cells did not survive prolonged culture in the combined absence of both amino acids (Figure 4A). Viable T cells activated for 72 hours under control conditions and in the absence of asparagine, but not Gln, were uniformly large blasting cells, as determined by analysis of forward scatter/side scatter parameters (Figure 4B). Furthermore, analysis of CellTrace Violet dilution after 72 hours of activation indicated that asparagine deprivation reduced but did not block antigen-stimulated T cell proliferation (Figure 4, C and D). By contrast, Gln deprivation completely prevented T cell proliferation in vitro.

Comparison of OT-I T cell phenotypes after 72 hours of peptide stimulation indicated that levels of CD25, CD71, and granzyme B expression were equivalent in control cells and cells activated in asparagine-deprived conditions (Figure 4, E–H). By contrast, T cells stimulated in the absence of Gln had very low levels or absent expression of cell surface activation markers and granzyme B (Figure 4, E–H). Furthermore, and in contrast to results from earlier time points, OT-I T cells activated for 72 hours in the absence of asparagine had unimpaired capacity to uptake glucose and long-chain fatty acids (Supplemental Figure 3, A and B), whereas levels of ATP production were comparable to control levels (Supplemental Figure 3C).

Furthermore, we tested the effects of initial T cell activation in the presence of extracellular asparagine and Gln followed by switching to media lacking asparagine and/or Gln. OT-I T cells were activated in IMDM for 24 hours, then switched to DMEM with or without asparagine and Gln for an additional 24 hours. Under all conditions, viable T cells had a blasting phenotype and expressed similar levels of CD71. Nonetheless, T cells transferred to Gln-free media had reduced viability and modestly reduced cell size and demonstrated impaired uptake of 2-NBDG (Supplemental Figure 3, D–H). By contrast, T cells transferred to asparagine-free media after initial activation in asparagine-replete conditions were unimpaired in these parameters.

We assessed the functional capacity of effector cytotoxic T lymphocytes (CTLs) differentiated for 6 days in the absence of either asparagine or Gln. OT-I T cells differentiated in the absence of Gln did not produce IFN-γ, and reduced proportions of cells were competent to produce TNF upon antigenic restimulation in nutrient-replete media (Figure 4, I and J). By contrast, CTLs differentiated in the absence of asparagine produced equivalent levels of effector cytokines upon restimulation in nutrient-replete media as compared with control cells (Figure 4, I and J). These data indicate that extracellular Gln but not asparagine is essential for CD8^+^ T cell differentiation to an inflammatory phenotype. We next sought to test whether there was any requirement for extracellular sources of asparagine in effector T cell function. To this end, we differentiated OT-I T cells in nutrient-replete IMDM media for 5 days, transferred to DMEM with or without asparagine prior to restimulation of the resultant CTLs in the presence or absence of asparagine. Asparagine deprivation did not impede effector T cell secretion of IFN-γ (Figure 4K).

### CD8^+^ T cells upregulate ASNS expression via the mTOR signaling pathway.

**Figure 5:**
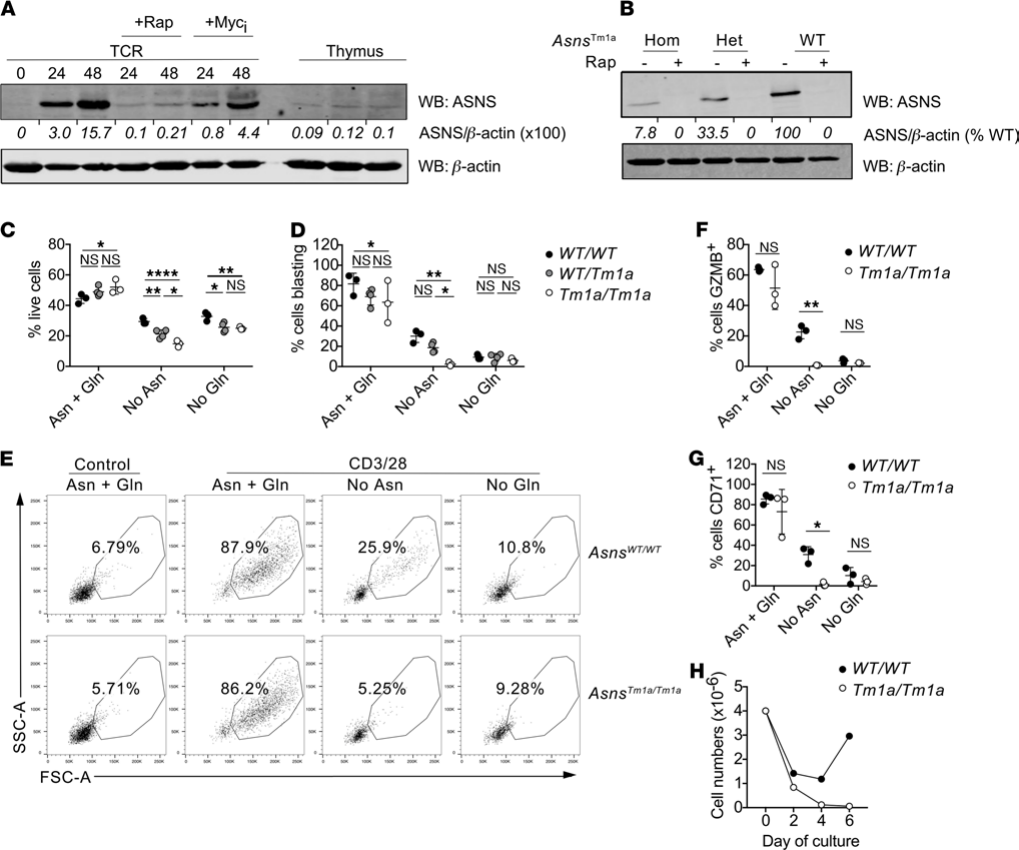
Asparagine synthetase expression enables T cell responses in asparagine-depleted conditions. (**A**) Western blots show asparagine synthetase (ASNS) protein levels in lysates of OT-1 T cells stimulated in IMDM with SIINFEKL peptide with or without rapamycin (rap) or Myc inhibitor (Myc_i_) for the indicated time periods, or thymocytes. (**B**) ASNS levels in lysates from activated lymph node T cell from homozygous, heterozygous, or WT *Asns^Tm1a^* mice. (**A** and **B**) β-Actin serves as a protein loading control. Values underneath blots represent relative ASNS expression levels (**A**) or ASNS levels as percentage of WT (**B**) calculated using the LI-COR Odyssey imaging system from 1 of 2 repeated experiments. Lymph node T cells from *Asns^Tm1a^* mice and controls were stimulated with anti-CD3/28 antibodies for 48 hours in DMEM with or without asparagine or Gln. Cell viability was assessed by exclusion of Live-Dead Aqua dye and flow cytometry (**C**). Proportions of gated live CD8^+^ T cells undergoing blastogenesis were assessed by FACS analysis of FSC-A/SSC-A parameters (**D** and **E**). Control cells were cultured in the presence of IL-7, which maintains cell viability without inducing T cell activation (**E**). Gated live CD8^+^ T cells were analyzed by FACS for levels of intracellular granzyme B (**F**) and cell surface CD71 (**G**). (**H**) Purified control and *Asns^Tm1a^* CD8^+^ T cells were activated in asparagine-free DMEM for 6 days. Live cells were enumerated throughout the time course of the experiment. (**C–H**) Data represent 1 of at least 3 repeated experiments, and individual dots represent technical replicates (*n* = 3). *P < 0.05, **P < 0.01, ****P < 0.0001, as determined by 2-way ANOVA with Sidak’s multiple comparisons tests.

We reasoned that the initial dependence of CD8^+^ T cells on extracellular asparagine could be a consequence of low/absent ASNS expression and that ASNS might be upregulated following T cell activation. To test this, OT-I T cells were activated for various time periods in nutrient-replete conditions and Western blot analysis of ASNS expression was performed. These data indicated that ASNS was not expressed in naive OT-I cells and at very low levels in thymocytes but was upregulated in response to TCR stimulation (Figure 5A and Supplemental Figure 4). Activation of the mTOR pathway and Myc transcription factor is central to metabolic reprogramming following T cell activation (23), whereas our data suggested that asparagine availability was rate limiting for mTOR activation at early stages of T cell activation (Figure 3). Western blots showed that treatment with rapamycin and Myc inhibitors substantially reduce TCR-induced upregulation of ASNS (Figure 5A and Supplemental Figure 4), suggesting that ASNS upregulation is a feature of mTORC1-dependent T cell metabolic reprogramming.

### T cell development and homeostasis are unimpaired in ASNS gene-trap mice.

To determine the role of ASNS expression in T cells, we assessed *Asns^Tm1a^* mice, a genetically modified mouse strain with a gene-trap insertion in intron 2 of the *Asns* locus. Previous studies of the role of ASNS in neural development determined that the gene-trap approach resulted in a hypomorphic *Asns* allele (24). Western blotting analysis of activated lymph node T cell lysates determined that homozygous expression of the hypomorphic allele resulted in a more than 90% decrease in ASNS protein expression, as compared with WT littermate controls, whereas heterozygous cells expressed intermediate levels (Figure 5B and Supplemental Figure 4). For all genotypes, TCR-induced ASNS expression was abolished by rapamycin (Figure 5B), consistent with the results from OT-I TCR transgenic experiments (Figure 5A). We next sought to determine whether ASNS deficiency impacted T cell developmental processes. Flow cytometry analysis of thymus and lymph node cells determined that homozygous *Asns^Tm1a^* mice had similar proportions and numbers of thymocyte populations and mature CD4^+^ and CD8^+^ T cells as compared with WT and heterozygous controls (Supplemental Figure 5, A–C). Furthermore, proportions of naive (CD44^lo^CD62L^hi^), central memory (CD44^hi^CD62L^hi^), and effector/effector memory (CD44^hi^CD62L^lo^) phenotype CD4^+^ and CD8^+^ T cells were similar in *Asns^Tm1a^* and control mice (Supplemental Figure 5D). These data indicate that homozygous expression of the hypomorphic *Asns* allele does not impinge on T cell development in the thymus nor on peripheral T cell homeostasis in a specific-pathogen free animal facility.

### ASNS expression is required for CD8^+^ T cell activation in the absence of extracellular asparagine.

To test the role for ASNS in T cell activation, we stimulated polyclonal lymph node cells from *Asns^Tm1a^* and control mice for 48 hours with plate-bound CD3/CD28 antibodies in DMEM media supplemented with or without Gln and/or asparagine. Similar to results using OT-I T cells, FACS analysis of polyclonal CD8^+^ T cells demonstrated impaired T cell viability in asparagine- and Gln-deprived conditions (Figure 5C). ASNS-deficient T cells had lower cell viability under conditions of asparagine deprivation than control T cells (Figure 5C). Similarly, TCR-induced CD8^+^ T cell blastogenesis was completely abrogated by the combination of genetic ASNS deficiency and extracellular asparagine deprivation (Figure 5, D and E). The combined absence of extracellular asparagine and ASNS deficiency also prevented TCR-induced upregulation of activation marker CD71 and granzyme B (Figure 5, F and G). Moreover, we assessed population expansion and survival of control and *Asns^Tm1a^* CD8^+^ T cells in asparagine-free media following 2 days of CD3/28 stimulation followed by a further 4 days of IL-2 stimulation. Although both control and *Asns^Tm1a^* cell numbers were reduced during the first 2 days of activation in asparagine-free conditions, viable control T cell numbers recovered during days 2–6 (Figure 5H), coincident with ASNS upregulation. By contrast, numbers of viable *Asns^Tm1a^* T cells were markedly impaired (Figure 5H). Taken together, these data suggest that T cell activation, metabolic reprogramming, and mTOR-dependent ASNS upregulation enable CD8^+^ T cells to function independently of extracellular sources of asparagine.

## Discussion

T cell activation is accompanied by, and dependent on, metabolic reprogramming and substantial upregulation of nutrient uptake. In the current work, we determined that, initially, antigen-stimulated CD8^+^ T cells require extracellular sources of the nonessential amino acid asparagine to maintain viability, to grow, and to initiate activation and metabolic remodeling. However, upon prolonged stimulation, CD8^+^ T cells diverge from a strict asparagine-dependent phenotype as a consequence of upregulation of ASNS expression. By contrast, Gln remains an essential amino acid for T cells throughout all stages of activation and differentiation.

The availability of nutrients is key to immune functionality. Gln is the most abundant amino acid in human serum, whereas the results of the current work confirm the findings of previous studies that Gln transporter and Gln uptake expression is critical for T cell activation (2). It had been assumed that lymphocytes are auxotrophic for asparagine through the interpretation of studies of the effect of bacterial asparaginases on transformed and nontransformed lymphocyte populations (13, 18, 19). Our results confirm that extracellular asparagine is required for initial stages of CD8^+^ T cell activation. In the absence of asparagine or Gln alone, TCR stimulation results in substantial levels of T cell death. Nonetheless, our data indicate that asparagine can partially compensate for the absence of Gln, and vice versa, as in the combined absence of both amino acids, TCR-stimulated T cells fail to survive. Similar results have been reported for transformed cells; in the absence of Gln, asparagine is required to maintain viability and proliferation of cancer cells, whereas high ASNS is associated with poor prognosis in several cancer types (25, 26).

Defective CD8^+^ T cell activation, growth, and metabolic reprogramming in asparagine-deprived conditions in vitro was associated with, and likely to be a consequence of, a reduced capacity for protein synthesis. Asparagine deprivation results in translational pausing at asparagine codons and decreased protein synthesis with a dominant effect on asparagine-rich proteins (27). In breast cancer cells, asparagine availability influences expression of proteins that regulate epithelial-mesenchymal transition, with subsequent effects on metastasis (14). Whether reduced asparagine availability effects specific subsets of proteins or has a more global effect on the T cell proteome remains to be investigated. Asparagine deprivation also resulted in decreased TCR-induced mRNA levels of the transcription factor *Tbx21* but not *Il2*. Therefore, asparagine deprivation can effect expression of proteins either through posttranscriptional effects alone or a combination of reduced transcription and protein synthesis. We anticipate that the major effect of asparagine deprivation on T cell activation is to limit protein synthesis but that there may also be indirect effects on transcription, perhaps as a consequence of diminished protein expression of the transcriptional machinery. An intriguing additional possibility is that asparagine availability regulates T cell activation by regulating the activity of the TCR proximal kinase Lck and subsequent downstream signals (28).

In addition to its fundamental role in protein synthesis, asparagine has been reported to function as an exchange factor for additional amino acids in tumor cells (29). Thus, intracellular asparagine levels control uptake of serine, arginine, and histidine and thereby controls mTORC1 activation and protein synthesis. Our experiments indicated that the availability of asparagine regulated TCR-induced mTORC1 activation, suggesting that asparagine may have a similar role as an amino acid exchange factor in T cells. An additional nonexclusive possibility is that asparagine deprivation may indirectly influence mTORC1 activation as a consequence of reduced expression of nutrient transporters and uptake of amino acids such as leucine. Consistent with this hypothesis, our data indicated that asparagine deprivation resulted in reduced TCR-induced expression of CD98 and capacity for uptake of kynurenine.

Our data have determined that effector CD8^+^ T cell populations have the capacity to function independently of extracellular asparagine, as a consequence of ASNS expression. These results also emphasize an additional role for Gln in T cell activation; in the absence of asparagine, Gln is required to serve as a nitrogen donor for the synthesis of asparagine via ASNS activity (13). Our analyses of *Asns* gene-trap mice strongly suggest that T cell development does not require ASNS expression. However, it is possible that residual expression of low levels of ASNS in *Asns^Tm1a^* mice, quantified as less than 10% of WT levels, is sufficient to enable T cell development or that extracellular asparagine levels in the thymus are sufficient for ASNS expression to be redundant. Indeed, the bioavailability of asparagine varies between distinct anatomical locations in vivo (14). Therefore, there are likely to be varying context-dependent requirements for ASNS expression on T cell development, homeostasis, and inflammatory responses in vivo. Wu and colleagues recently reported that, in mice fed an asparagine-free diet, T cell homeostasis was largely unimpaired while CD8^+^ T cell responses to *Listeria monocytogenes* infection and B16 melanoma were impeded, as compared with mice receiving asparagine (28). Those data are consistent with an important role for asparagine uptake in T cell activation in vivo and with previous reports of the immunosuppressive effects of asparaginases (19), with the caveat that asparaginases frequently have glutaminase activity. Nonetheless, it is unclear at present when and where extracellular asparagine is limiting under physiological conditions and what role ASNS upregulation plays in CD8^+^ T cell activation in vivo. Similarly, the role for ASNS and asparagine uptake in CD4^+^ T cell activation and differentiation, as well as in other immune cell populations, remains to be explored. Future analysis of the impact of lineage-specific deletion of ASNS in the T cell lineage and in vivo analyses of ASNS-deficient T cell function will resolve this outstanding question. Nonetheless, our data show that the low levels of ASNS expression in peripheral *Asns^Tm1a^* CD8^+^ T cells are insufficient to maintain TCR-induced activation in the complete absence of extracellular asparagine in vitro.

It is important to note that ASNS activity generates both glutamate (Glu) and asparagine, and depletes Gln and aspartate. The extent to which intracellular Glu levels are affected by ASNS deficiency is not clear. In this regard, Glu is taken up from the extracellular environment by activated T cells, and intracellular levels of both Gln and Glu increase substantially in T cells activated in nutrient-replete conditions (8). Nonetheless, our experiments suggested that *Asns^Tm1a^* T cell responses were not impaired when extracellular asparagine was available, suggesting that the effect of ASNS deficiency on T cell activation was unlikely to be a consequence of reduced Glu levels. It should be noted that these experiments depended on the use of DMEM medium, which is relatively low in glucose (4 mM) and was supplemented with dialyzed serum, to control Gln and asparagine levels. Under such conditions, control T cell responses were suboptimal, therefore, it remains possible that ASNS expression is rate limiting for optimal T cell responses in nutrient-replete conditions.

In summary, our work has shown that activated T cells coordinate nutrient uptake with upregulated expression of key regulators of intracellular amino acid biosynthesis. Elevated ASNS expression via the mTORC1 and Myc pathways is part of a transcriptional program that endows T cells with the capacity to maximize their nutrient resources during effector immune responses.

## Methods

### Mice.

*Asns^Tm1a(EUCOMM)/Wtsi^* (referred to herein as *Asns^Tm1a^*) gene-trap mice were generated by the International Mouse Phenotyping Consortium and were imported to the University of Leeds St. James’s Biomedical Services (SBS) animal facility from MRC Harwell (Harwell, United Kingdom). *Asns^Tm1a^* mice were backcrossed with in-house C57BL/6J mice; in all experiments, littermates and/or age-matched in-house bred C57BL/6J mice were used as controls. OT-I *Rag1^–/–^* (30) mice were maintained in the SBS facility. Where applicable, group sizes are indicated by individual data points.

### Cell culture and stimulation.

OT-I TCR transgenic or polyclonal T cells were obtained from lymph nodes and/or spleens of OT-1 *Rag1^–/–^* or *Asns^Tm1a^* and control mice, respectively. For experiments investigating the role of extracellular amino acids, cells were cultured in DMEM containing 4 mM glucose (Gibco) supplemented with 5% dialyzed FBS (Gibco), penicillin-streptomycin (Gibco), 50 μM 2-mercaptoethanol (Gibco) with or without 2 mM L-Gln and 300 μM anhydrous L-asparagine (both Sigma-Aldrich). Otherwise, cells were cultured in IMDM (Gibco) containing 25 mM glucose, 5% FBS, 50 μM 2-mercaptoethanol, 4 mM L-Gln, and 190 μM freebase L-asparagine. OT-I T cells were stimulated with 10^-8^ M SIINFEKL peptides. For generation of OT-I CTLs, after 2 days of culture, cells were washed and cultured for a further 4 days in media containing 20 ng/mL recombinant human IL-2 (Peprotech). For analysis of effector cytokine production, T cells were restimulated in media containing 10^-9^ M SIINFEKL and Brefeldin A (2.5 μg/mL, Sigma-Aldrich). For activation of polyclonal T cells, mixed LN cells were activated using plate-bound anti-CD3ε (1.5 μg/mL) (2C11, BioLegend) and soluble anti-CD28 (37.51, BioLegend, 1 μg/mL) as indicated, for2 days, then transferred to media containing 20 ng/mL IL-2. In some experiments, CD8^+^ T cells were purified by negative selection and magnetic sorting (Miltenyi Biotech) prior to activation as previously described. To assess the role of the mTORC1 and Myc pathways in regulating ASNS expression, cells were stimulated in the presence or absence of 100 nM rapamycin (Tocris Bioscience) or Myc inhibitor 10058-F4 (Sigma-Aldrich).

### Flow cytometry and antibodies.

The following antibodies were used: CD4-allophycocyanin (APC) or -brilliant violet 421 (BV421) (clone GK1.5), CD8β-phycoerythrin-Cy7 (clone YTS156.7.7), CD25-PE (clone PC61.5), CD36-PE (clone HM36), CD44-APC-Cy7 (clone 1M7), CD69-peridinin-chlorophyll-protein Cy5.5 (PerCP Cy5.5) (clone H1.2F3), CD71-FITC (clone RI7217), CD98-Alexa Fluor 647 (clone 4F2), TCRαβ-FITC (clone H57-597), IFN-γ–AF488 (clone XMG1.2), TNF-PerCP Cy5.5 (clone MP6-XT22), granzyme B–Pacific blue (clone GB11), and Tbet-PE (clone 4B10) (all BioLegend) and eomesodermin-AF488 (eBioscience). Unconjugated anti-rpS6 phospho-Ser240/244 (clone D68F8, Cell Signaling Technology) was counterstained with goat anti-rabbit secondary reagents (Molecular Probes). Live/dead aqua and Zombie NIR dyes were from Life Technologies and BioLegend, respectively. For intracellular staining, cells were fixed in Phosflow fix buffer (BD Pharmingen) or eBioscience FoxP3 fix/permeabilization buffers prior to staining in permeabilization buffers. For analysis of protein synthesis, cells were labeled with OPP (20 μM, Stratech Scientific) and the Click-iT Plus AF488 picolyl azide kit (Life Technologies) according to the manufacturers’ instructions. Cycloheximide (100 μg/mL, Sigma-Aldrich) was added 15 minutes prior to labeling as a negative control. For analysis of nutrient uptake, T cells were cultured with 2-deoxy-2-([7-nitro-2, 1, 3-benzoxadiazol-4-yl]amino)-glucose (2-NBDG) (50 μM for 1 hour, Abcam), BODIPY-C16, 1 μM, for 30 minutes, Invitrogen) or kynurenine (800 μM in HBSS buffer for 4 minutes, Sigma-Aldrich), and washed 3 times in PBS. For BODIPY and kynurenine uptake, cells were counterstained with Zombie NIR prior to FACS analysis. Samples were acquired with LSR II (BD) or Cytoflex LX (Beckman Coulter) flow cytometers, and data were analyzed using FlowJo software (Treestar).

### ELISA.

To prevent consumption of secreted IL-2, CD25 blocking antibodies (clone 3C7, BioLegend) were added to culture media prior to T cell stimulation. Cytokine levels in culture supernatants were assessed using mouse IL-2 and IFN-γ DuoSet ELISA kits (R&D Systems), according to the manufacturers’ instructions.

### Quantitative RT-PCR.

OT-I T cells were activated in DMEM with or without asparagine as described, and cell pellets (1.5–2 × 10^6^) stored at –80°C until RNA preparation. RNA was prepared using Qiagen RNeasy Mini Kits and cDNA synthesized using Superscript III RT and oligo(dT)20 primers (Life Technologies). The relative expression of *Rpl13a* was used to normalize gene expression across samples. *Il2* and *Tbx21* expression was quantified by qRT-PCR using the ddCt method and Taqman reagents (Taqman Universal Mastermix II, Applied Biosystems) using the QuantStudio 7 Real-Time PCR system (Applied Biosystems). The following Taqman probes were used: *Il2* — Mm00434256_m1; *Tbx21* — Mm00450960_m1; *Rpl13a* — Mm05910660_g1. Samples were amplified using a 2-step cycling method (40 cycles: 95°C for 15 seconds, 60°C for 1 minute).

### Metabolic analyses.

ECAR and OCR values were acquired using a Seahorse XFe96 Analyzer, as described previously (31). Briefly, stimulated T cells were plated (10^5^ viable cells/well) in Cell-Tak (Corning) coated Seahorse Analyzer XFe96 culture plates in Seahorse assay media supplemented with glucose (10 mM), Gln (2 mM), and pyruvate (1 mM). Data were collected in Wave software and analyzed using GraphPad Prism software. For Mito Stress tests, oligomycin (1 μM), FCCP (1.5 μM), and rotenone/antimycin A (0.5 μM) were injected using the standard XFe96 protocol.

### ATP assay.

T cells were stimulated as indicated in figure legends. For the measurement of cellular ATP levels, a luminescence-based kit (Luminescent ATP Detection Assay Kit, Abcam, ab113849) was used according to the manufacturer’s instructions.

### Western blotting.

T cells were stimulated as indicated in figure legends, and cell lysates prepared in RIPA buffer. Protein concentration of lysates was assessed by Bradford Assay (Thermo Fisher Scientific), and 20 μg protein per sample loaded on polyacrylamide gels. Samples were separated by SDS-PAGE, and proteins transferred to nitrocellulose membranes (Bio-Rad). Membranes were blocked in LI-COR blocking buffer (LI-COR Biosciences). Primary antibodies (rabbit anti-ASNS – HPA029318, Atlas Antibodies, mouse anti–β-actin, clone AC-15, Sigma-Aldrich) were diluted in LI-COR blocking buffer and detected using goat anti-rabbit-AF680 and goat anti-mouse AF790 secondary reagents (Molecular Probes). Protein bands were detected and quantified using a Li-COR Odyssey Imaging System.

### Statistics.

Two-tailed Student’s *t* tests, Mann-Whitney test, and 1- or 2-way ANOVA with Tukey’s or Sidak’s multiple comparison tests were performed using GraphPad Prism software. *P* values or adjusted *P* values of less than 0.05 were considered significant. In graphs, dots represent values from replicate samples, and error bars represent SDs. Numbers of experimental and/or technical replicates are described in figure legends.

### Study approval.

All mouse breeding and experiments were approved by and subject to the conditions of a UK Home Office Project License (PDAD2D507) held by RJS and were reviewed by the University of Leeds Animal Welfare and Ethics Board.

## Author contributions

HCH and RJS designed the research. HCH performed the majority of experiments. HCH, RJB, CMF, RJS, and LS acquired and analyzed the data. RJB, CMF, RJS, and LS performed experiments. RJS wrote the manuscript. All authors read and discussed manuscript drafts.

## Supplementary Material

Supplemental data

## Version 1 04/06/2021

In-Press Preview

## Version 2 05/10/2021

Electronic publication
