# Supplementary material for: Coordination of asparagine uptake and asparagine synthetase expression modulates CD8+ T cell activation
Source: JCI Insight. 2021 May 10;6(9):e137761. doi: 10.1172/jci.insight.137761 (PMC8262305; doi:10.1172/jci.insight.137761)
Supplement: Supplemental data [file jciinsight-6-137761-s244.pdf]

Coordination of asparagine uptake and asparagine synthetase expression modulates CD8$^+$ T cell activation

Helen Carrasco Hope,[1] Rebecca J. Brownlie,[1] Christopher M. Fife,[1] Lynette Steele,[1] Mihaela Lorger[1] and Robert J. Salmond[1]

[1]Leeds Institute for Medical Research at St James's, University of Leeds, Wellcome Trust Brenner Building, St James's University Hospital, Leeds, United Kingdom

Supplemental Figures 1-5

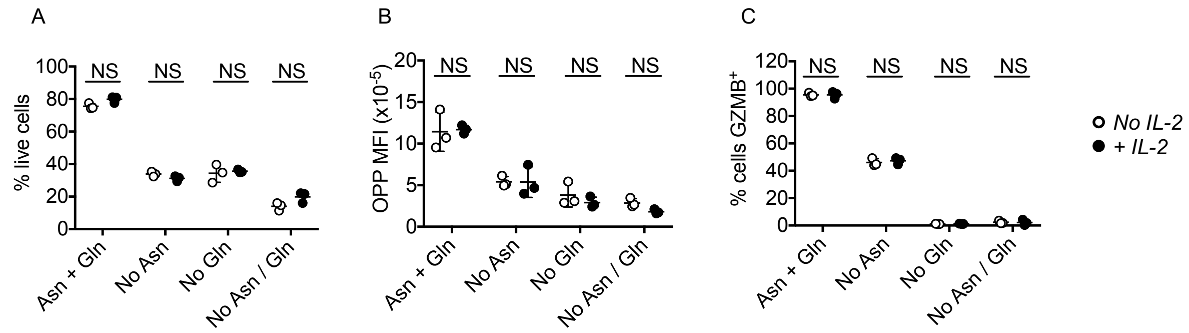

Supplemental Figure 1: Exogenous IL-2 does not rescue T cell activation in amino acid-deprived conditions

OT-1 T cells were activated for 24h (A, B) or 48h (C) in DMEM ± Asn and/or Gln, in the presence or absence of 1ng/mL recombinant IL-2. Proportions of live cells (A) were determined by live-dead aqua dye exclusion and FACS. Levels of protein synthesis were assessed by incorporation of OPP, Click chemistry labelling and FACS (B). Intracellular granzyme B expression was assessed by FACS (C). NS – not significant as determined by 2-way ANOVA.

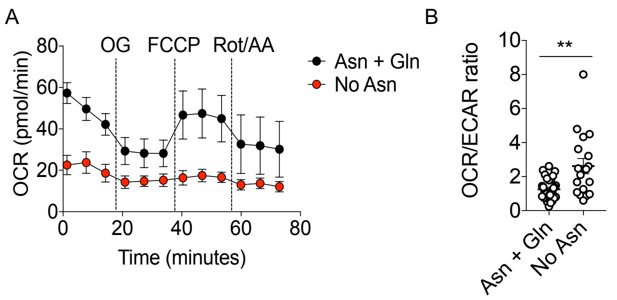

Supplemental Figure 2: Reduced mitochondrial function in Asn-deprived CD8$^+$ T cells

OT-I T cells were activated with SIINFEKL peptide for 24h in DMEM ± Asn. (A) Oxygen consumption rates (OCR) were measured using the Seahorse XFe96 analyser, using a Mitostress^{TM} kit. OG – oligomycin, FCCP- Carbonyl cyanide-4-(trifluoromethoxy)phenylhydrazone. Rot/AA – rotenone/antimycin A. Values are mean OCR readings from replicate wells (n=6) ± SD. (B) Ratio for the basal OCR/extracellular acidification rates (ECAR) rates for cells stimulated in the presence or absence of Asn (dots represent technical replicates, n=30). ** - p<0.01 by Student's *t*-test. Data represent one of two repeated experiments.

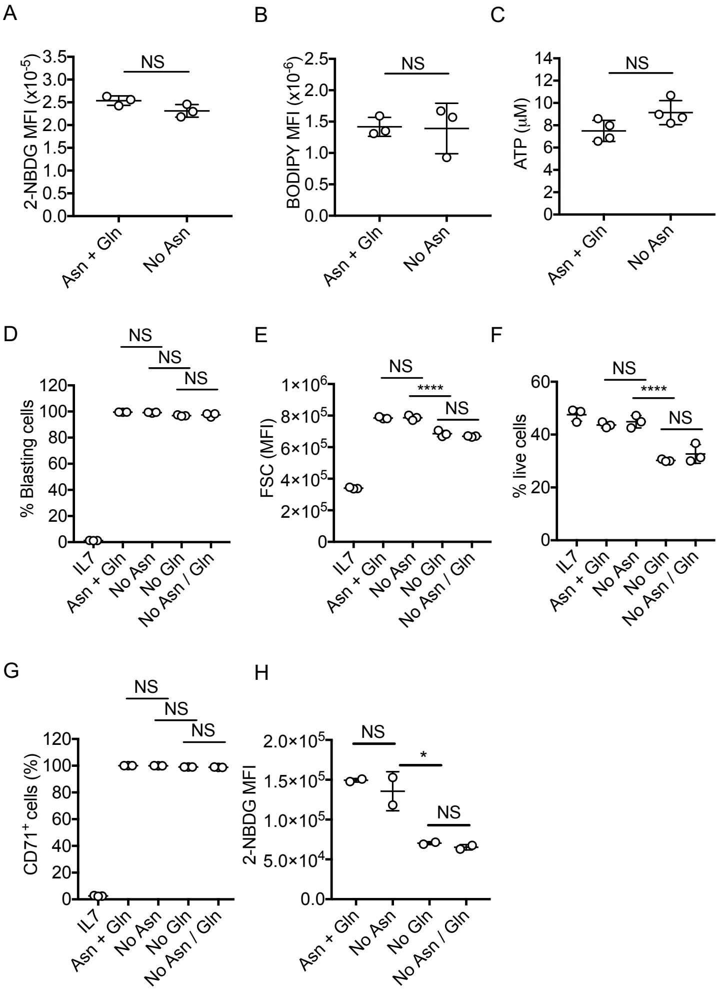

Supplemental Figure 3: T cells lose dependence upon extracellular Asn upon prolonged activation

OT-1 T cells were activated for 72h with SIINFEKL peptide in DMEM ± Asn (A-C). Uptake of fluorescent 2-NB-d-glucose (A) and BODIPY™-C16 (B) was assessed by FACS. Data shown are mean fluorescence intensities (MFI). (C) Cellular ATP levels were assessed using a luminescent assay. (D-H) OT-I T cells were stimulated with SIINFEKL peptide in complete IMDM for 24h, then were switched to DMEM ± Asn ± Gln for 24h. Proportions of blasting cells (A), cell size (FSC) (B), viability (C), CD71 expression (D) and 2-NBDG uptake (E) by OT-I T cells after 48h of stimulation were assessed by flow cytometry. Data are representative of 3 repeated experiments, dots represent technical replicates (for part C n=4, otherwise n=3). NS – not significant, * $p<0.05$, **** $p<0.001$ as assessed by Mann-Whitney test (A-C) or by one-way ANOVA with Tukey's multiple test (D-H).

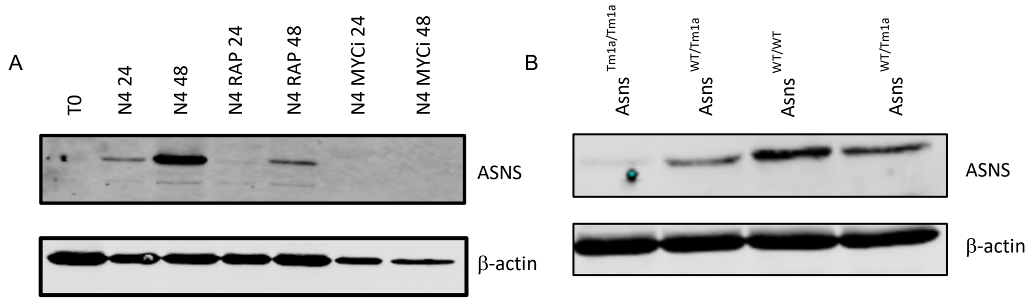

Supplemental Figure 4: Replicate western blotting experiments for ASNS expression

A) OT-1 T cells were activated for 0-48h with SIINFEKL (N4) peptide in the presence of rapamycin (RAP) or Myc inhibitor (MYCi). B) Levels of ASNS in activated $Asns^{Tm1a}$ gene-trap T=lymph node T cells. Data show western blots of ASNS in cell lysates; β actin serves as a loading control.

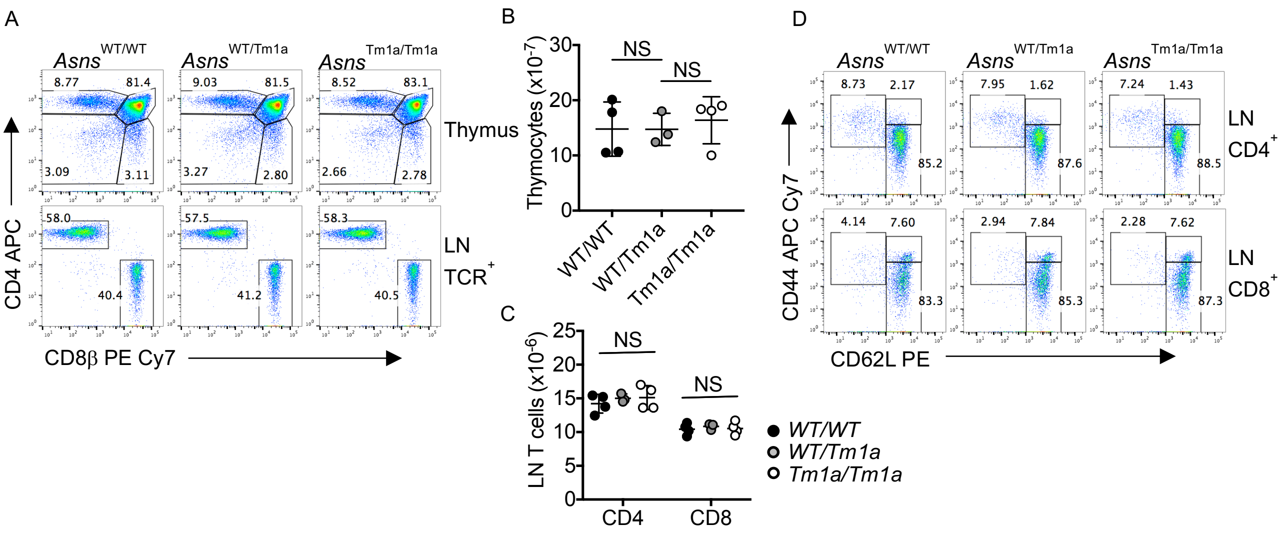

Supplemental Figure 5: T cell development and homeostasis are unimpeded in Asns^{Tm1a} mice

(A) Thymocyte and lymph node (LN) cell populations from littermate Asns^{Tm1a} mice were analysed by flow cytometry. Total thymocytes (B) and LN CD4 and CD8 T cells (C) were enumerated from 3-4 age-matched male mice of each genotype. (D) Proportions of naïve, effector and memory cells within gated CD4^{+} and CD8^{+}T cell populations were analysed by analysis of CD44 and CD62L expression by flow cytometry. FACS dotplots are representative of 5-6 mice of each genotype. Values on dotplots represent % cells within the defined gates. NS – not significant, as determined by one-way ANOVA
